# Frequency of apical periodontitis in root‐filled teeth restored with post and core: A 5‐year retrospective study

**DOI:** 10.1002/cre2.881

**Published:** 2024-05-26

**Authors:** Louise Johansson, Jakob Jonsson Sjögren, Anders Wirén, Alf Eliasson, Fredrik Frisk

**Affiliations:** ^1^ Dental Research Department Örebro University Örebro Sweden; ^2^ Specialist Clinic of Endodontology Public Dental Health Service Region Örebro County Sweden; ^3^ Department of Endodontics Malmö University Malmö Sweden; ^4^ Clinical Epidemiology and Biostatistics Örebro University Örebro Sweden; ^5^ Department of Endodontology Institute for Postgraduate Dental Education Jönköping Sweden; ^6^ School of Health and Welfare Jönköping University Jönköping Sweden; ^7^ Department of Endodontology, Institute of Odontology, Sahlgrenska Academy University of Gothenburg Gothenburg Sweden

**Keywords:** endodontics, periapical periodontitis, post and core technique, root canal therapy

## Abstract

**Background:**

In conjunction with post placement in root‐filled teeth with periapical healthy conditions, root canal retreatment may be performed to improve the seal of the root canal. Whether root canal retreatment for technical reasons (retreatments in teeth without apical periodontitis (AP)) results in lower frequency of AP is unknown.

**Objective:**

To examine whether there is a difference in frequency of AP between roots with root canals retreated for technical reasons, and roots with root canals not retreated before post placement, with a minimum follow‐up of 5 years. Also, to examine changes in root filling quality following root canal retreatment for technical reasons.

**Methods:**

This retrospective study included radiographs of 441 root‐filled roots without periapical radiolucencies at baseline, scheduled for post and core treatment. Follow‐up data for a minimum of 5 years were available for 305 roots (loss to follow‐up 30.8%), 46 of which were retreated for technical reasons. Two calibrated observers assessed root filling sealing quality and length, respectively, and periapical status according to the Periapical Index. The main outcome of the study, AP, was used as the dependent variable and all analyses were performed at root level.

**Results:**

The overall frequency of AP at follow‐up was 13.8%. The difference in frequency of AP between retreated (4.3%) and nonretreated (15.4%) root canals was not statistically significant, *p* = .061. Analyses including only roots with preoperatively inadequate root filling quality showed a statistically significant difference (*p* = .017) between the two treatment groups (2.4% *vs*. 22.9%).

**Conclusions:**

Root canal retreatment for technical reasons before post and core placement significantly reduces the frequency of AP in roots with inadequate root filling quality.

## 1 INTRODUCTION

When restoring root‐filled teeth, a post and core may be required to achieve retention for coronal restoration. According to previous studies, the presence of a post per se will not affect the prevalence of apical periodontitis (AP) in previously root‐filled teeth (Kvist et al., 1989; Tronstad et al., 2000; Tsesis et al., 2013). In endodontic treatment, the purpose of the root filling is to seal the root canal properly to prevent oral microorganisms and nutritional elements from maintaining an existing infection or re‐infecting the root canal. It is well established that the technical quality of root fillings, assessed radiographically with regard to length and lateral seal, is associated with periapical status (Frisk et al., 2008; Kirkevang et al., 2000; Sjögren et al., 1990). Also, Kvist et al. (1989) reported that roots with posts where the remaining root filling was <3 mm showed a significantly higher frequency of periapical radiolucency compared with roots where the root filling was ≥3 mm. In conjunction with post and core placement, the root filling may be exposed to oral microorganisms. It is reasonable to assume that a technically inadequate root filling is less efficient in preventing ingress of oral microorganisms into the root canal than a technically adequate root filling.

In their clinical study, Bergenholtz et al. (1979) investigated whether retreatment of root canals, with or without periapical radiolucencies, resulted in improved technical quality of the root fillings. Retreatments carried out based on technical indications only remained a healthy periapical status in most cases. However, the study did not provide a comparison with teeth with root fillings that were not retreated. Thus, whether root canal retreatment for technical reasons (retreatments in teeth without AP) really results in lower frequency of AP is unknown. The purpose of retreating the root canal before post placement is preventive to reduce the risk of developing AP, and therefore the risk for prosthetic failure or the need for a potentially complicated surgical procedure.

The aims of this study were twofold: (1) To examine whether there is a difference in frequency of AP between roots with root canals retreated for technical reasons, and roots with root canals that were not retreated, before post placement, with a minimum follow‐up of 5 years. (2) To examine changes in root filling quality following root canal retreatment for technical reasons.

## 2 MATERIAL AND METHODS

### 2.1 Study design and cohort

**Figure 1:**
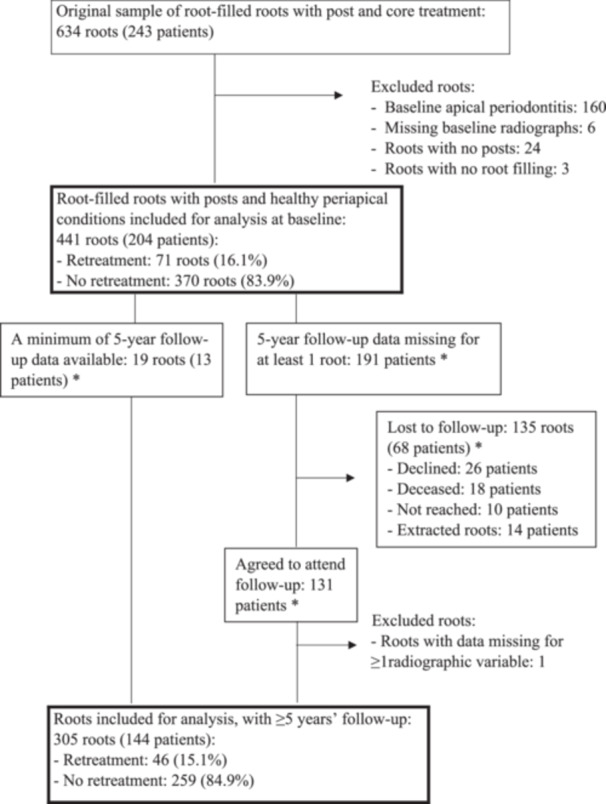
Flow chart of the original number of root‐filled roots with post and core treatment, the number of patients and the reasons for exclusion. *Note that a patient may have one root with follow‐up and another root that was lost to follow‐ up. Therefore, the number of patients represented by the total number of roots, when split into roots with and without follow‐up data, may appear inflated.

In this retrospective study, data were collected from all patients with one or more root‐filled teeth treated with post and core treatment at the Department of Prosthetic Dentistry in Örebro, Sweden, between July 2008 and December 2011. The patients were identified from a database of dental records applying specific codes for all types of post and core treatment, a search which resulted in 243 patients with 634 roots in 511 root‐filled teeth, treated with gold or cobalt‐chromium alloy cast posts. All other types of posts were excluded from this study. Periapical radiographs taken at the time of post placement were missing in four teeth, leaving 507 teeth with complete baseline data. After retrospective assessment of the existing baseline radiographs, with data collection at root level, all roots assessed with periapical radiolucencies were excluded, leaving 441 roots in 204 patients without periapical radiolucencies for baseline analysis (Figure 1).

Data were collected by two dentists at the Departments of Endodontic and Prosthetic Dentistry in Örebro during 2017–2018. Patients (*n* = 191) for whom a 5‐year radiographic follow‐up was missing for at least one root were invited to a free‐of‐charge radiographic examination of the current root or roots. In addition, data were extracted from dental records and radiographs.

The following variables were registered: the patient's gender, age at post placement, tooth and root type, the dentist's level of education, and root filling status. Root filling status was recorded as: previously root‐filled root (≥1 year before post placement), recently root‐filled root (<1 year before post placement) or retreated root canal; root filling quality before root filling retreatment and after post and core placement; periapical status after a minimum of 5 years; and follow‐up time.

In total, 144 patients with 305 root‐filled roots met the inclusion criteria for follow‐up analysis. One hundred and thirty‐one patients agreed to attend the radiographic follow‐up examination. Figure 1 presents a flow chart of the sample. Twenty‐six patients declined participation, 18 patients were deceased, ten patients could not be reached by letter or telephone, and in 14 patients the current root or roots were extracted within the 5‐year follow‐up.

### 2.2 Ethical considerations

The study design was approved by the regional Ethics Committee at Uppsala University, Uppsala, Sweden (dnr 2016/180).

### 2.3 Radiographic examination

An intraoral periapical image with all visible apices was taken of the current root or roots. All intraoral digital radiographs were produced using an intra‐oral X‐ray machine (ProX or Intra; Planmeca Oy, Helsinki, Finland) at 8 mA, 60 kVP, and with optimized time exposure using a phosphor dental plates system and laser scanners (Digora® Optime imaging plate system; Soredex Corporation, Helsinki, Finland) with image acquisition software (LifeCare Dental Vision v2.0, Tieto Corporation Healthcare Welfare, Espoo, Finland). The images were transferred to a dedicated radiology workstation (Eizo monitors, RX240; resolution 166 ×1200; Eizo Corporation, Ishikawa, Japan) where assessment was performed in a dark room without daylight.

### 2.4 Radiographic assessment and variables

Periapical radiographs taken at the time of post placement were selected for baseline assessment; radiographs taken a minimum of 5 years after placement of the post were selected for follow‐up assessment. The radiographic assessment included the root filling and the periapical status. The assessment of the root filling consisted of measuring the distance between the post and root filling, the length of the remaining root filling, the distance between the root filling and radiographic apex, and assessment of the sealing quality. The sealing quality of the root filling was assessed as adequate if there were no visible voids lateral or apical to the root filling, and the root filling appeared homogeneous. All measurements were made to the nearest 0.1 mm and were then categorized as presented in Table 1. The variables length (the distance between the root filling and radiographic apex) and sealing quality were merged into the variable root filling quality. Root filling quality was dichotomized into adequate root filling quality (adequate sealing quality and root filling ending 0–2 mm from apex) and inadequate root filling quality (root fillings deviating from this criterion). The radiographic assessment of periapical status was performed according to the Periapical Index (PAI) (Ørstavik et al., 1986); PAI scores (1–5) were dichotomized into healthy periapical status (1–2) and AP (3–5). The categorization of all radiographic variables is presented in Table 1.

**Table 1:** Variables recorded on periapical radiographs.

Variable	Categories
Distance between post and root filling Length of the remaining root filling	No distance ≤0.1 mm Distance >0.1 mm Adequate ≥3 mm Inadequate <3 mm
Distance between root filling and radiographic apex	>2 mm from apex 0–2 mm from apex Overfilling (root filling material beyond radiographic apex)
Sealing quality of root filling Root filling quality	Adequate sealing = root filling is homogeneous without voids lateral or apical to the root filling Inadequate sealing = root filling is not homogeneous and/or voids lateral or apical to the root filling Adequate quality = adequate sealing + root filling ending 0–2 mm from apex Inadequate quality = root filling deviating from the criteria listed for adequate quality
Periapical Index (PAI) (Ørstavik et al., 1986)	1 normal periapical structures 2 small changes in bone structure 3 changes in bone structure with some mineral loss 4 periodontitis with well‐defined radiolucent area 5 severe periodontitis with exacerbating features

In addition to the previously described assessment procedure, all roots that were root canal‐retreated before placement of the post were also assessed regarding root filling length (the distance between the root filling and radiographic apex) and sealing quality before root filling retreatment. This radiographic assessment consisted of measuring the distance between the root filling and the radiographic apex, and the sealing quality of the root filling.

### 2.5 Observer calibration

Before determining the periapical status, the two observers were individually calibrated to the PAI, by observing a set of 100 reference radiographs with different periapical expressions, in accordance with Ørstavik et al. (1986) Observer agreement with the PAI was calculated and presented with Cohen's kappa.

Regarding root filling length and sealing quality, a calibration was made between the two observers by together observing randomly chosen radiographs of root‐filled teeth and discussing their findings, to create a reference for the assessment. Then the two observers independently assessed 30 randomly chosen radiographs from the study material with regard to root filling length and sealing quality. Interobserver agreement was calculated and presented with Cohen's kappa.

After calibration, the two observers independently examined all radiographs. In case of interobserver disagreement, the observers discussed their findings until they reached consensus.

### 2.6 Statistical methods

Descriptive statistics were used to analyse patient and root‐specific characteristics. Bivariate analyses with chi‐square tests were performed to analyse differences in frequencies of root filling status among categories of the other independent variables. Such differences could possibly confound the association between the main outcome, AP, and the treatment variable. For analysing paired observations regarding change in root filling quality following root canal retreatment, McNemar's test was used.

The main outcome of the study, AP, was used as the dependent variable in subsequent statistical analyses. Periapical status was indicated by PAI score, dichotomized into 0 = no AP (PAI score 1–2) and 1 = AP (PAI score 3–5). All analyses were performed at root level. Since some patients contributed more than one root and/or tooth to the study, there was a certain likelihood that some roots would be at greater risk of developing AP than others, largely because they were from the same tooth and/or patient. To adjust for these potential intra‐individual correlations, a mixed effects logistic model was used for analysis. The statistical model included individual‐ and tooth‐level variables as random effects and root‐level variables as fixed effects, and estimated outcome correlations between roots of the same tooth and/or the same patient. As these random effects, for both the main analysis including 305 roots and the additional analysis including 159 roots, were either negligible or nonsignificant (data not shown), they were dropped from the model and a binary logistic statistical model with only fixed effects was used instead. To adjust the analysis for potential confounding factors, independent variables that showed a significant association with the outcome in a univariate analysis were included in the final, multivariate model, together with the main predictor variable, retreatment.

The level of significance was set at 0.05 and odds ratios (ORs) and corresponding 95% confidence intervals (CIs) were calculated.

Inter‐ and intra‐observer agreement was assessed using Cohen's kappa (Cohen, 1960). Data were analysed using SPSS, version 27.0 (IBM Corporation, Armonk, NY).

## 3 RESULTS

### 3.1 Descriptive data

Out of 441 root‐filled roots with posts that met the inclusion criteria for this study, follow‐up data for a minimum of 5 years were available for 305 roots (loss to follow‐up 30.8%). The mean follow‐up time was 78 months (standard deviation [SD] 12.5; range 60–130). The follow‐up analysis included 144 patients, 68 women (47.2%) and 76 men (52.8%), mean age 60.4 years (SD 12.9; range 15–86), with one or more root‐filled roots with posts. Of the 305 root‐filled roots with posts, 39.7% were premolars, 38.7% were incisors or canines, and 21.6% were molars. Table 2 presents patient and root characteristics.

**Table 2:** Demographic data for the 144 patients and root characteristics for the 305 roots (268 teeth) included in the follow‐up analysis in the study, and demographic data for the 68 patients lost to follow‐up. Note that a patient may have one root that was followed up and another root that was lost to follow‐up.

Patients *n *= 144		Loss to follow‐up patients *n *= 68
Mean age, years (SD)	60.4 (12.9)	61.9 (13.6)
Median age, years (range)	62.5 (15–86)	65.0 (16–85)
Gender		
Female, *n* (%)	68 (47.2)	38 (55.9)
Men, *n* (%)	76 (52.8)	30 (44.1)
Teeth in study per individual, *n* (%)		
1	76 (52.8)	
2	38 (26.4)	
3	17 (11.8)	
4	7 (4.9)	
5	2 (1.4)	
6	3 (2.1)	
9	1 (0.7)	
Mean (SD)	1.90 (1.3)	
Roots in study per individual, *n* (%)		
1	68 (47.2)	
2	36 (25.0)	
3	22 (15.3)	
4	6 (4.2)	
5	6 (4.2)	
6	2 (1.4)	
7	2 (1.4)	
9	1 (0.7)	
10	1 (0.7)	
Mean (SD)	2.12 (1.6)	
Roots (*n *= 305)		
Tooth type		
Incisor or canine, *n* (%)	118 (38.7)	
Premolar, *n* (%)	121 (39.7)	
Molar, *n* (%)	66 (21.6)	
Follow‐up, months		
Mean (SD)	78 (12.5)	
Median (range)	76 (60–130)	
Root filling status		
No retreatment, *n* (%)	259 (84.9)	
Retreatment, *n* (%)	46 (15.1)	
Dentist's level of education		
Specialist prosthodontist, *n* (%)	224 (73.4)	
Postgraduate dentist, *n* (%)	81 (26.6)	
Distance between post and root filling		
No distance, *n* (%)	32 (10.5)	
Distance (>0.1 mm), *n* (%)	273 (89.5)	
Length of remaining root filling		
Adequate (≥3 mm), *n* (%)	270 (88.5)	
Inadequate (<3 mm), *n* (%)	35 (11.5)	
Distance between root filling and apex		
>2 mm from apex, *n* (%)	91 (29.8)	
0–2 mm from apex, *n* (%)	191 (62.6)	
Overfilling, *n* (%)	23 (7.5)	
Sealing quality		
Adequate, *n* (%)	250 (82.0)	
Inadequate, *n* (%)	55 (18.0)	

SD = standard deviation.

There were 142 (46.6%) previously (defined as ≥1 year before post and core treatment) and 117 (38.4%) recently ( < 1 year before post and core treatment) root‐filled roots, and 46 (15.1%) retreated root fillings (defined as retreated, adjacent to post placement, for technical reasons). Since the analysis showed the same frequency of AP at follow‐up (15.4%) between the two former groups they were merged into one “no retreatment” group.

Of the 441 root‐filled roots with posts analysed at baseline, root filling retreatment before placement of the post had been performed in 71 roots (16.1%). Within the variable tooth type, molars (38.0%) and premolars (38.0%) were the most common teeth for retreatment.

### 3.2 Observer variation

Intra‐observer agreement with the PAI was assessed by calculating Cohen's kappa (0.70 for both observers). Regarding root filling length and sealing quality, interobserver agreement gave a kappa of 0.81 for length and 0.72 for sealing quality.

### 3.3 Frequency of apical periodontitis

In the total material (305 roots) the frequency of AP was 13.8% (42 roots) at a minimum of 5 years' follow‐up. Roots treated with root canal retreatment before placement of the post had a frequency of AP in 4.3% and roots with root canals that were not retreated had a frequency of AP in 15.4% of cases, at follow‐up. A univariate logistic regression model including only root filling status as an independent variable showed that the difference in frequency of AP at follow‐up between the two groups was not statistically significant (OR 0.249, *p* = .061, 95% CI 0.058–1.068). Two other independent variables, distance between root filling and apex, and sealing quality, showed a significant association with AP in the univariate analysis, as described below. The main predictor of interest, retreatment, was therefore included in a multivariate logistic regression along with these two variables to adjust for any confounding effect they might have on the possible association between root filling status and AP.

Retreatment showed a similar effect on AP in this analysis, but was also not statistically significant (OR 0.286, *p* = .099, 95% CI 0.065–1.265) (Table 3, Supplementary Tables S1 and S2). However, a sub‐analysis using the chi‐square test revealed that retreated and nonretreated roots were not comparable regarding several characteristics before retreatment (Table 4). The majority of the retreated root canals were assessed to have had inadequate root filling quality before retreatment, 91.1% (41 roots), compared with 45.6% (118 roots) of the roots with no retreatment. Therefore, all roots with inadequate root filling quality that were retreated and all those not retreated were analysed separately to estimate the frequency of AP at follow‐up (Table 5, Supplementary Table S1). The analysis showed that roots with inadequate root filling quality that had been retreated before post and core treatment had a statistically significantly lower frequency of AP at follow‐up (OR 0.084, *p* = .017, 95% CI 0.011–0.642), compared with roots with inadequate root filling quality that were not retreated. The same analyses on roots with adequate root filling quality could not be made because of too few roots (four in total) in the group with retreated root fillings.

**Table 3:** Logistic regression analysis of associations between independent variables and the dependent variable AP in 305 root‐filled roots after  ≥ 5 years. Beta values, standard errors and degrees of freedom are presented in Supplementary Tables S1 and S2.

Variable	No AP, *n* (%)	AP, *n* (%)	Univariate analysis	*p*‐value	Multivariate analysis	*p*‐value
			OR (95% CI)		OR (95% CI)	
Age						
15–55 years	48 (84.2)	9 (15.8)	Reference			
56–65 years	124 (85.5)	21 (14.5)	0.903 (0.386–2.111)	0.814		
66–86 years	91 (88.3)	12 (11.7)	0.703 (0.277–1.787)	0.459		
Gender						
Female	130 (86.1)	21 (13.9)	Reference			
Male	133 (86.4)	21 (13.6)	0.977 (0.510–1.875)	0.945		
Follow‐up period						
60–75 months	126 (82.9)	26 (17.1)	Reference			
76–130 months	137 (89.5)	16 (10.5)	0.566 (0.290–1.104)	0.095		
Operator						
Specialist prosthodontist	195 (87.1)	29 (12.9)	Reference			
Postgraduate dentist	68 (84.0)	13 (16.0)	1.285 (0.632–2.615)	0.488		
Tooth type						
Incisor or canine	100 (84.7)	18 (15.3)	Reference			
Premolar	106 (87.6)	15 (12.4)	0.786 (0.376–1.644)	0.523		
Molar	57 (86.4)	9 (13.6)	0.877 (0.370–2.081)	0.766		
Root filling status						
No retreatment	219 (84.6)	40 (15.4)	Reference			
Retreatment	44 (95.7)	2 (4.3)	0.249 (0.058–1.068)	0.061	0.286 (0.065–1.265)	0.099
Distance between post and root filling						
No distance	27 (84.4)	5 (15.6)	Reference			
Distance (>0.1 mm)	236 (86.4)	37 (13.6)	0.847 (0.307–2.337)	0.748		
Length of remaining root filling						
Adequate (≥3 mm)	234 (86.7)	36 (13.3)	Reference			
Inadequate (<3 mm)	29 (82.9)	6 (17.1)	1.345 (0.522–3.465)	0.540		
Distance between root filling and apex						
0–2 mm from apex	166 (86.9)	25 (13.1)	Reference			
>2 mm from apex	82 (90.1)	9 (9.9)	0.729 (0.325–1.633)	0.442	0.586 (0.247–1.388)	0.225
Overfilling	15 (65.2)	8 (34.8)	3.541 (1.362–9.208)	0.009	4.677 (1.714–12.765)	0.003
Sealing quality						
Adequate	224 (89.6)	26 (10.4)	Reference			
Inadequate	39 (70.9)	16 (29.1)	3.535 (1.738–7.186)	<0.001	4.853 (2.232–10.551)	<0.001

Abbreviations: AP, apical periodontitis; CI, confidence interval; OR, odds ratio.

**Table 4:** Distribution of 304 root‐filled roots according to treatment and each independent variable, referring to preoperative status.

Variable	Retreatment, *n* (%)	No retreatment, *n* (%)	p‐value^a^
Gender			
Women	24 (53.3)	126 (48.6)	0.562
Men	21 (46.7)	133 (51.4)	
Tooth type			
Incisor or canine	11 (24.4)	107 (41.3)	0.012
Premolar	17 (37.8)	102 (39.4)	
Molar	17 (37.8)	50 (19.3)	
Distance between root filling and apex			
Adequate (0–2 mm)	10 (22.2)	167 (64.5)	<0.001
Inadequate ( > 2 mm or overfilling)	35 (77.8)	92 (35.5)	
Sealing quality of root filling			
Adequate	21 (46.7)	211 (81.5)	<0.001
Inadequate	24 (53.3)	48 (18.5)	
Root filling quality			
Adequate	4 (8.9)	141 (54.4)	<0.001
Inadequate	41 (91.1)	118 (45.6)	

*Note*: Total *n* = 305 roots (100%); one root was excluded because of missing data for one or more radiographic variables.

^a^ Chi‐square test.

**Table 5:** Periapical status in 159 roots with inadequate preoperative root filling quality, after  ≥ 5 years. Logistic regression analysis was applied. Data were missing for 65 roots (29%). Beta values, standard errors and degrees of freedom are presented in Supplementary Table S3.

Variable	No AP, *n* (%) AP, *n* (%)		Univariate analysis OR (95% CI)	*p*‐value
Age				
15–55 years	27 (84.4)	5 (15.6)	Reference	
56–65 years	60 (78.9)	16 (21.1)	1.440 (0.478–4.335)	0.517
66–86 years	44 (86.3)	7 (13.7)	0.859 (0.248–2.980)	0.811
Follow‐up period				
60–75 months	62 (78.5)	17 (21.5)	Reference	
76–130 months	69 (86.3)	11 (13.8)	0.581 (0.253–1.337)	0.202
Dentist's level of education				
Specialist in prosthodontics	104 (85.2)	18 (14.8)	Reference	
Postgraduate dentist	27 (73.0)	10 (27.0)	2.140 (0.886–5.166)	0.091
Tooth type				
Incisor or canine	39 (75.0)	13 (25.0)	Reference	
Premolar	54 (84.4)	10 (15.6)	0.556 (0.221–1.396)	0.211
Molar	38 (88.4)	5 (11.6)	0.395 (0.128–1.215)	0.105
Root filling status				
No retreatment	91 (77.1)	27 (22.9)	Reference	
Retreatment	40 (97.6)	1 (2.4)	0.084 (0.011–0.642)	0.017

Abbreviations: AP, apical periodontitis; CI, confidence interval; OR, odds ratio.

### 3.4 Associations between independent variables and frequency of apical periodontitis

A binary logistic regression model was used to investigate potential associations between the independent variables and AP after a minimum of 5 years' follow‐up (Table 3). Roots with overfillings were statistically significantly associated with a higher frequency of AP at follow‐up in both the univariate (OR 3.541, *p* = .009, 95% CI 1.362–9.208) and the multivariate analysis (OR 4.677, *p* = .003, 95% CI 1.714–12.765) compared with the reference (roots with root fillings ending 0–2 mm from apex). In addition, roots with inadequate root filling sealing quality were also statistically significantly associated with a higher frequency of AP at follow‐up (univariate analysis: OR 3.535, *p* < .001, 95% CI 1.738–7.186; multivariate analysis: OR 4.853, *p* < .001, 95% CI 2.232–10.551) compared with roots with adequate root filling sealing quality.

Age, gender, follow‐up period, operator, tooth type, distance between post and root filling, and length of the remaining root filling were not statistically significantly associated with frequency of AP at follow‐up.

### 3.5 Root filling quality following retreatment

Before placement of the post, the root canal was retreated in 71 roots. Out of these, 58 (89.2%) were assessed to have inadequate root filling quality before retreatment. After retreatment, inadequate root filling quality was found in 30 roots (46.2%). Root canal retreatment significantly improved the technical quality of the root filling regarding both root filling length and sealing quality (*p* < .001) (Table 6).

**Table 6:** Root filling length, sealing quality (adequate = root filling is homogeneous without voids lateral or apical to the root filling) and root filling quality (adequate = adequate sealing + root filling ending 0–2 mm from apex) before and after retreatment based on technical indication. Total *n* = 71 roots (100%). Roots (*n* = 6) with data missing for one or more radiographic variables were excluded.

Variable	Before root filling retreatment, *n* (%)	After root filling retreatment, *n* (%)	*p*‐value^a^
Distance between root filling and radiographic apex, *n *= 66			
0–2 mm from apex	16 (24.6%)	42 (63.6%)	<0.001
>2 mm from apex, and/or	49 (75.4%)	24 (36.4%)	
overfilling			
Sealing quality, *n*=65			
Adequate	30 (46.2%)	56 (86.2%)	<0.001
Inadequate	35 (53.8%)	9 (13.8%)	
Root filling quality, *n* = 65			
Adequate	7 (10.8%)	35 (53.8%)	<0.001
Inadequate	58 (89.2%)	30 (46.2%)	

^a^ McNemar's test.

## 4 DISCUSSION

This study reports the frequency of AP in roots with root canals retreated for technical reasons, and roots with nonretreated root canals, before post and core placement, with a minimum follow‐up of 5 years.

The results showed that the odds for AP at follow‐up were almost twelve times higher (OR 0.084, *p* = .017) for roots with preoperatively inadequate filling quality that were not retreated compared with root canals that were retreated before post placement (22.9% *vs*. 2.4%). During post preparation procedures, the coronal seal of the tooth is broken, a substantial amount of the previous root filling is removed, and the apical seal of the root canal may be jeopardized. Regardless of previous root filling material or technique, contamination of the root canal can occur after a short period of exposure to saliva (Siqueira, 2001). Therefore, the results of this study support the assumption that, when exposed to the oral environment, a technically inadequate root filling may not prevent the root canal from being infected as effectively as a technically adequate root filling, in conjunction with post and core treatment. In the present study only indirect post and core treatments were included due to different treatment procedure compared to direct post and core treatments. The included teeth were also the cohort for a prosthetic study including only gold or cobalt‐chromium alloy cast posts.

Kvist et al. (1989) suggested that the remaining root filling must not be shorter than 3 mm since these roots showed a significantly higher frequency of periapical radiolucencies compared with roots where the root filling was ≥3 mm, in their study. In vitro studies have shown that a remaining apical root filling of 5–7 mm prevents leakage better than one of 3 mm (Metzger et al., 2000). In the present study, the length of the remaining root filling was not statistically significantly associated with frequency of AP at follow‐up. The majority of roots, 88.5%, had a remaining root filling of ≥3 mm, leaving only 11.5% with shorter measurements.

Nor was a longer distance between root filling and apex ( > 2 mm) associated with periapical status at follow‐up. The fact that we did not find significant differences within these variables may be due to there having been no real differences or due to the small groups for analysis in this material, but more likely it is explained by the fact that all roots in our study had healthy periapical status at baseline.

Roots with inadequate sealing quality of the remaining root filling had a statistically significantly higher frequency of AP at follow‐up compared with roots with adequate root filling sealing quality. In addition, roots with overfillings were statistically significantly associated with a higher frequency of AP at follow‐up in both univariate and multivariate analysis compared with the reference, roots with root fillings ending 0–2 mm from apex. These results are in agreement with previous clinical and epidemiological studies (Frisk et al., 2008; Sjögren et al., 1990). A clinical study by Bergenholtz et al. (1979) showed that root fillings with technical shortcomings were, following retreatment, improved with regard to length and lateral seal. Retreatments carried out for technical reasons only were successful (remained healthy periapical status) in 94% of cases, after a 2‐year follow‐up. The remaining 6% of the treated teeth developed AP during the follow‐up period, mainly associated with overinstrumentation and overfilling. Thus, the study showed that when retreatment is performed for technical reasons and the instruments are kept within the root canal, the prognosis for maintaining healthy periapical conditions may be considered good. In agreement with this, we found that root canal retreatment in the present study significantly improved the technical quality of the root filling regarding both root filling length and sealing quality. Moreover, the frequency of AP at follow‐up, after retreatment was low, 4.3%.

Overall, in our study, roots with root canal retreatment before placement of the post had AP at follow‐up in 4.3% of the cases. Roots with root fillings that were not retreated had AP at follow‐up in 15.4% of the cases. The difference in frequency of AP at follow‐up was not statistically significant in neither the univariate (*p* = .061) nor the multivariate analysis (*p* = .099). This result may be explained by the small number of roots in the group of retreated roots. Also, a sub‐analysis revealed that the two groups of root‐filled roots receiving different treatments (retreatment or no retreatment) were not comparable regarding root characteristics before root canal retreatment in this material.

Out of the 71 roots where root canal retreatment was performed, 89.2% of the root fillings were assessed as inadequate before root canal retreatment. It was remarkable that out of all roots with root fillings that were not retreated before post placement, many (45%) of the root fillings were assessed as inadequate. Inadequate root filling quality is a well‐known predictor for AP (Laukkanen et al., 2021; Ng et al., 2008) and should be considered in the preoperative analysis of the tooth before post and core treatment. An observational study by Petersson et al. (1991) examined changes in periapical status of root‐filled teeth over time, with a follow‐up period of 11 years. The results showed that previously root‐filled teeth with healthy periapical conditions developed AP in 14% of cases during the observational period. The incidence of AP was significantly higher for teeth with inadequate root filling quality (23%) than in teeth with adequate root filling quality (4%). On the other hand, Kirkevang et al. (2007) found that the incidence of AP in root‐filled teeth at 6 years' follow‐up was not statistically significantly associated with root filling quality. However, their study showed correlations between inadequate root filling quality and persistent AP. The results in the present study regarding frequency of AP at follow‐up in roots with inadequate root filling quality that were not retreated before post placement (22.9%) are in agreement with the findings reported by Petersson et al. (1991) The mean time of follow‐up in our study was 78 months, with a range of 60–130 months, but no statistically significant association was found between follow‐up time and frequency of AP.

The results showed an overall AP frequency of 13.8% at follow‐up. A recent retrospective radiographic study reported an incidence of AP of 6.9% in 102 root‐filled teeth treated with post and core treatment, with a minimum follow‐up of 8 months (Haereid et al., 2022). In that study, undergraduate or postgraduate students conducted endodontic treatment under controlled and standardized conditions. These circumstances, in combination with the relatively short follow‐up, may explain the lower incidence of AP.

As it is retrospective, our study has certain sources of bias. There is a risk of selection bias due to loss to follow‐up. However, based on the analysis of nonattenders, no major differences between attenders and nonattenders could be seen regarding age and gender. Another potential source of selection bias is the fact that each operator decided whether to retreat the root filling or not without adherence to specific clinical guidelines pertaining to retreatment of root fillings for technical reasons. Also, the study was conducted on patients referred to and treated at the Department of Prosthetic Dentistry in Örebro. Consequently, the results may not be generalizable to Swedish patients in general dental care. The observer agreement with PAI was substantial for both observers. Regarding root filling quality the inter observer agreement was substantial for both root filling length and seal.

The results of this study support previous findings that a root filling that ends within the root and presents adequate sealing quality is associated with a lower frequency of AP. Our study also demonstrates that roots with root fillings with inadequate root filling quality that were retreated before post placement had a significantly lower frequency of AP at follow‐up compared with roots that were not retreated. However, the operator needs to assess the potential need for root canal retreatment before commencing prosthodontic treatment, regardless of technical quality of the existing root filling assessed radiographically. The purpose of this preventive treatment is to reduce the risk of developing AP to reduce the risk for prosthetic failure or the need for a potentially complicated surgical procedure. Tooth type and position in the jaw, as well as some medical conditions, may limit the surgical options. In those cases, the benefit of a preventive measure is clear even though root canal retreatment is unnecessary in a substantial fraction of patients.

## 5 CONCLUSIONS

Within the limitations of this study, we may conclude that root canal retreatment for technical reasons before placement of cast post and core significantly decreases the frequency of AP in roots with inadequate root filling quality. Also, root canal retreatment resulted in improved technical quality of the root fillings regarding both length and sealing quality.

## AUTHOR CONTRIBUTIONS

Louise Johansson contributed to conception and design of the study, performed data collection and drafted the manuscript. Fredrik Frisk contributed to conception and design of the study and to manuscript drafting. Alf Eliasson contributed to conception and design of the study. Anders Wirén and Jakob Jonsson Sjögren performed data analysis and tabulation. All authors critically revised the manuscript and approved the final version.

## CONFLICT OF INTEREST STATEMENT

The authors declare no conflicts of interest.

## Supporting information

Supporting information.

Supporting information.

## Data Availability

The data that support the findings of this study are available on request from the corresponding author. The data are not publicly available due to privacy or ethical restrictions.
