# Supplementary material for: Frequency of apical periodontitis in root‐filled teeth restored with post and core: A 5‐year retrospective study
Source: Clin Exp Dent Res. 2024 May 26;10(3):e881. doi: 10.1002/cre2.881 (PMC11128751; doi:10.1002/cre2.881)
Supplement: Supplementary file 1 — Supporting information. [file CRE2-10-e881-s001.docx]

| TABLE S1 Univariate logistic regression analysis of associations between independent variables and the dependent variable AP in 305 root-filled roots after ≥5 years’ follow-up. Presents supplementary data regarding beta values, standard errors and degrees of freedom. | | | | | | | | |  |  |  |
| --- | --- | --- | --- | --- | --- | --- | --- | --- | --- | --- | --- |
| Variable | No AP, *n* (%) AP, *n* (%) | | Univariate analysis  OR (95% CI) | p-value | β | | SE (β) | DF | | |  |
| Age  15–55 years  56–65 years  66–86 years  Gender  Female  Male  Follow-up period  60–75 months  76–130 months  Operator  Specialist prostho- dontist  Postgraduate dentist  Tooth type  Incisor or canine  Premolar  Molar  Root filling status  No retreatment  Retreatment  Distance between post and root filling  No distance  Distance (>0.1 mm)  Length of remaining root filling  Adequate (≥3 mm)  Inadequate (<3 mm)  Distance between root filling and apex  0–2 mm from apex  >2 mm from apex  Overfilling  Sealing quality  Adequate  Inadequate | 48 (84.2)  124 (85.5)  91 (88.3)  130 (86.1)  133 (86.4)  126 (82.9)  137 (89.5)  195 (87.1)  68 (84.0)  100 (84.7)  106 (87.6)  57 (86.4)  219 (84.6)  44 (95.7)  27 (84.4)  236 (86.4)  234 (86.7)  29 (82.9)  166 (86.9)  82 (90.1)  15 (65.2)  224 (89.6)  39 (70.9) | 9 (15.8)  21 (14.5)  12 (11.7)  21 (13.9)  21 (13.6)  26 (17.1)  16 (10.5)  29 (12.9)  13 (16.0)  18 (15.3)  15 (12.4)  9 (13.6)  40 (15.4)  2 (4.3)  5 (15.6)  37 (13.6)  36 (13.3)  6 (17.1)  25 (13.1)  9 (9.9)  8 (34.8)  26 (10.4)  16 (29.1) | Reference  0.903 (0.386–2.111)  0.703 (0.277–1.787)  Reference  0.977 (0.510–1.875)  Reference  0.566 (0.290–1.104)  Reference  1.285 (0.632–2.615)  Reference  0.786 (0.376–1.644)  0.877 (0.370–2.081)  Reference  0.249 (0.058–1.068)  Reference  0.847 (0.307–2.337)  Reference  1.345 (0.522–3.465)  Reference  0.729 (0.325–1.633)  3.541 (1.362–9.208)  Reference  3.535 (1.738–7.186) | 0.814  0.459  0.945  0.095  0.488  0.523  0.766  0.061  0.748  0.540  0.442  0.009  <0.001 | -0.102  -0.352  -0.023  -0.569  0.251  -0.241  -0.131  -1.391  -0.167  0.296  -0.316  1.265  1.263 | 0.433  0.476  0.332  0.341  0.362  0.376  0.441  0.743  0.518  0.483  0.411  0.488  0.362 | | 1  1  1  1  1  1  1  1  1  1  1  1  1 | |  | |

AP = apical periodontitis; CI = confidence interval; DF = degree of freedom; OR = odds ratio; SE = standard error.

| TABLE S2 Multivariate logistic regression analysis of associations between independent variables and the dependent variable AP in 305 root-filled roots after ≥5 years’ follow-up. Presents supplementary data regarding beta values, standard errors and degrees of freedom. | | | | | | |
| --- | --- | --- | --- | --- | --- | --- |
| Variable | No AP, *n* (%) AP, *n* (%) | | Multivariate analysis  OR (95% CI) | p-value | β | SE (β) DF |
| Root filling status  No retreatment  Retreatment  Distance between root filling and apex  0–2 mm from apex  >2 mm from apex  Overfilling  Sealing quality  Adequate  Inadequate | 219 (84.6)  44 (95.7)  166 (86.9)  82 (90.1)  15 (65.2)  224 (89.6)  39 (70.9) | 40 (15.4)  2 (4.3)  25 (13.1)  9 (9.9)  8 (34.8)  26 (10.4)  16 (29.1) | Reference  0.286 (0.065–1.265)  Reference  0.586 (0.247–1.388)  4.677 (1.714–12.765)  Reference  4.853 (2.232–10.551) | 0.099  0.225  0.003  <0.001 | -1.251  -0.534  1.543  1.580 | 0.758 1  0.440 1  0.512 1  0.396 1 |

AP = apical periodontitis; CI = confidence interval; DF = degree of freedom; OR = odds ratio; SE = standard error.
