# Supplementary material for: Frequency of apical periodontitis in root‐filled teeth restored with post and core: A 5‐year retrospective study
Source: Clin Exp Dent Res. 2024 May 26;10(3):e881. doi: 10.1002/cre2.881 (PMC11128751; doi:10.1002/cre2.881)
Supplement: Supplementary file 2 — Supporting information. [file CRE2-10-e881-s002.docx]

| TABLE S3 Periapical status in 159 roots with inadequate preoperative root filling quality, after ≥5 years’ follow-up. Logistic regression analysis was applied. Data are missing for 65 roots (29%). Presents supplementary data regarding beta values, standard errors and degrees of freedom. | | | | | | |  |
| --- | --- | --- | --- | --- | --- | --- | --- |
| Variable | No AP, *n* (%) AP, *n* (%) | | Univariate analysis  OR (95% CI) | p-value | β SE (β) | DF |  |
| Age  15–55 years  56–65 years  66–86 years  Follow-up period  60–75 months  76–130 months  Operator’s level of education  Specialist prosthodontist  Postgraduate dentist  Tooth type  Incisor or canine  Premolar  Molar  Root filling status  No retreatment  Retreatment | 27 (84.4)  60 (78.9)  44 (86.3)  62 (78.5)  69 (86.3)  104 (85.2)  27 (73.0)  39 (75.0)  54 (84.4)  38 (88.4)  91 (77.1)  40 (97.6) | 5 (15.6)  16 (21.1)  7 (13.7)  17 (21.5)  11 (13.8)  18 (14.8)  10 (27.0)  13 (25.0)  10 (15.6)  5 (11.6)  27 (22.9)  1 (2.4) | Reference  1.440 (0.478–4.335)  0.859 (0.248–2.980)  Reference  0.581 (0.253–1.337)  Reference  2.140 (0.886–5.166)  Reference  0.556 (0.221–1.396)  0.395 (0.128–1.215)  Reference  0.084 (0.011–0.642) | 0.517  0.811  0.202  0.091  0.211  0.105  0.017 | 0.365 0.562 1  -0.152 0.635 1  -0.542 0.425 1  0.761 0.450 1  -0.588 0.470 1  -0.930 0.573 1  -2.474 1.036 1 | |  |
| AP = apical periodontitis; CI = confidence interval; DF = degree of freedom; OR = odds ratio; SE = standard error. | | | | | | |  |
